# Digging into the low molecular weight peptidome with the OligoNet web server

**DOI:** 10.1038/s41598-017-11786-w

**Published:** 2017-09-15

**Authors:** Youzhong Liu, Sara Forcisi, Marianna Lucio, Mourad Harir, Florian Bahut, Magali Deleris-Bou, Sibylle Krieger-Weber, Régis D. Gougeon, Hervé Alexandre, Philippe Schmitt-Kopplin

**Affiliations:** 1 UMR PAM Université de Bourgogne/Agrosup Dijon, Institut Universitaire de la Vigne et du Vin, Jules Guyot, Rue Claude Ladrey, Dijon, France; 2 Research Unit Analytical BioGeoChemistry, Department of Environmental Sciences, Helmholtz Zentrum München, Ingolstädter Landstr. 1, Neuherberg, Germany; 3 Technische Universität München, Chair of Analytical Food Chemistry, Freising-Weihenstephan, Germany; 4 Lallemand SAS, 19 rue des Briquetiers, Blagnac, France

## Abstract

Bioactive peptides play critical roles in regulating many biological processes. Recently, natural short peptides biomarkers are drawing significant attention and are considered as “hidden treasure” of drug candidates. High resolution and high mass accuracy provided by mass spectrometry (MS)-based untargeted metabolomics would enable the rapid detection and wide coverage of the low-molecular-weight peptidome. However, translating unknown masses (<1 500 Da) into putative peptides is often limited due to the lack of automatic data processing tools and to the limit of peptide databases. The web server OligoNet responds to this challenge by attempting to decompose each individual mass into a combination of amino acids out of metabolomics datasets. It provides an additional network-based data interpretation named “Peptide degradation network” (PDN), which unravels interesting relations between annotated peptides and generates potential functional patterns. The *ab initio* PDN built from yeast metabolic profiling data shows a great similarity with well-known metabolic networks, and could aid biological interpretation. OligoNet allows also an easy evaluation and interpretation of annotated peptides in systems biology, and is freely accessible at https://daniellyz200608105.shinyapps.io/OligoNet/.

## Introduction

Oligopeptides are polymers composed of 2 to 20 amino acids linked by amide bonds. Various types of natural bioactive oligopeptides have been discovered, displaying tremendous diversity in structure and function. Such compounds play critical roles in regulating biological processes e.g. growth factors^1^, neurotransmitters^2^, hormone^3^, toxins^4^, antibiotics^5^ etc. Natural oligopeptides are gaining considerable biotechnological and pharmaceutical importance. For instance, the tripeptide glutathione, found in fruits and vegetables, has become a popular dietary supplement to prevent oxidative damage to cells^6^. Other studies showed that the human peptidome, the cleavage product of larger proteins is a rich but unexplored “hidden treasure” of disease-specific biomarkers and drug candidates^7^. The discovery of natural bioactive oligopeptides has been widely studied in recent years. To detect new and unexpected biomarkers, an untargeted peptidomics approach is often applied to characterize the entire oligopeptidic profile. Such studies usually start with the purification and the extraction of the peptidome (peptides~0.4–12 kDa). The peptide-enriched samples are then analyzed by tandem mass spectrometry (MS/MS)^8, 9^ so that the most abundant peptides are identified by matching fragment masses with predicted MS/MS spectra of “digested” protein sequences in the database (Mascot^10^, MaxQuant^11^ and others).

In addition to untargeted peptidomics, oligopeptidic biomarkers have been reported from MS-based untargeted metabolomic studies^12, 13^. By investigating the complete set of low-molecular-weight metabolites (majorly <1 500 Da) in a biological system^14^, untargeted metabolomics, especially high resolution MS-based, can cover a part of the peptidome that consists of shorter oligopeptides^15^. Indeed, untargeted metabolomics could be a fast, simple and efficient alternative to exploit the low-molecular-weight (LMW) peptidome. Firstly, minimal sample preparation is required compared to peptidomics, which introduces less bias and allows studying the peptides along with other important metabolites. Moreover, the high sensitivity and mass accuracy of high-field MS-techniques used for metabolic profiling could reveal a tremendous number of potential oligopeptides in different matrices, even at very low concentrations^12, 13, 16, 17^. However, these potential peptide features have rarely been characterized in previous studies.

Typically, several thousands of mass features could be detected in a full-scan based MS screening in the *m/z* range of 100–1 500^18^. Thus, studying the LMW peptidome by untargeted metabolomics requires the rapid identification of potential peptide features via automatic data processing. In other words, suppose an unknown mass signal (e.g. 780.475 Da) is detected, how do we decide whether the signal could be an oligopeptide without knowing its fragments? Searching the *m/z* value in a metabolite library seems to be a straightforward solution. However, such databases contain a limited number of oligopeptides since the number of possible amino acid combinations could easily reach millions with increased peptide length^19^. For instance, the METLIN database^20^ currently contains di-, tri- and some of tetra- and penta-peptides, whereas the 100–1 500 Da mass range could cover longer peptides (e.g. the MW of the heptapeptide β-casein f169-175 is 780.475 Da^21^). In addition, these databases are usually limited to peptides that consist of proteinogenic amino acids. A database-independent solution to annotate any potential oligopeptides is to decompose the *m/z* value into a combination(s) of amino acids. Böcker *et al*.^22^ has related this task to the well-known Money Changing Problem (MCP) and developed an efficient algorithm called DECOMP. Their web server (http://bibiserv2.cebitec.uni-bielefeld.de/decomp) can decompose thousands of masses in a short time. We have tested several metabolomics datasets on their server. Based on the results, each mass signal from an untargeted MS screening can be annotated to: i) unique amino acid combination (UAAC) ii) multiple amino acid combinations (MAAP) or iii) no possible combination.

UAAC mass signals are interesting targets for LMW peptidome studies. Firstly, their sequences could be easily validated by fragmentation and de novo sequencing^8^. Furthermore, by studying the amino acid difference between two UAAC signals (sequenced or not), we could generate interesting biochemical hypotheses. For instance, the mass signal composed of two prolines and two alanines (noted as “A2P2”) has a difference of two alanines with the mass signal “P2”. Since metabolomics provides a static snapshot of metabolites in cells or biofluids, the coexistence of both signals might indicate an oligopeptidase activity involved in the hydrolysis of oligopeptides into amino acids^23–26^. In eukaryote cells, the degradation of oligopeptides is considered as the final step of the recycling of proteasome products^23^. For microorganisms, the breakdown of oligopeptides by extracellular proteolytic system plays an important role in generating utilizable nitrogen source for cell growth^25, 26^.

To generate maximal hypotheses from data, we propose a novel system biology approach called the “Peptide degradation network” (PDN) based on the functional Kendrick analogous mass difference network (fMDN)^27^. In the fMDN, two nodes (two mass signals) are connected if their mass difference (edge between nodes) agrees with one of user-defined repetitive functional units. In the PDN, to mimic the peptide degradation, we restrict the functional units to the loss of one or a combination of amino acids. Therefore, each node represents a UAAC mass signal, and a higher MW signal (e.g. A2P2) is connected to a lower one (e.g. P2) only if it contains all its amino acids.

The OligoNet web server that we developed allows rapid annotation of UAAC and MAAP signals from untargeted metabolomics data, followed by the PDN construction, visualization and network structure analysis. As a complementary study, UAAC signals are annotated in the KEGG database^28^ and visualized in metabolic pathways along with other putative metabolites in the dataset. We illustrate how the web server aids biological interpretation of untargeted metabolomics data with a concrete example.

## Program description

### Implementation details

OligoNet is a web server developed in R. On the server-side, *m/z* values from MS screening experiments are first decomposed into a combination(s) of user-defined amino acid. The closest related MCP can be resolved in two ways on OligoNet: i) using a recursive function written entirely in R, ii) using the third-party web server DECOMP^22^. For the second solution, metabolomic data are first converted to the JSON format and requests to DECOMP are performed using the *Rcurl* library^29^. The directed PDN is built as an *igraph* object^30^ based on all identified UAAC signals. OligoNet uses the *visNetwork* package for the network visualization. The *KEGGREST* library is used for the mass annotation in the KEGG database and for the visualization of metabolic pathways. The client-side interface was developed using R Shiny framework.

### Upload metabolomics data

The required metabolomics data (File 1) for OligoNet is a mass peak list from high-field MS experiments (neutral masses or positive/negative m/z). The first line of the file must contain a header with desired column names. The dataset has to be organized as follows:

i. First column: unique IDs assigned for each mass signal.
ii. Second column: a list of experimental or theoretical masses without any adducts, such as Na, K, Br and Cl. It is not recommended to include masses higher than 1 600 Da. Masses should be rounded to the same decimal places.
iii. Third column: the intensity of each mass signal (in case of direct-infusion MS) or the area of their corresponding chromatograph peaks (in case of LC/GC-MS).
iv. More columns (optional): users could add more intensities or areas from the 4th column if multiple samples are available. It is recommended to provide at least 10 samples for a better evaluation of correlations between network nodes.

User could add all additional information about mass signals in File 2 (optional), such as the retention times, statistical scores and p-values. This information will be displayed when visualizing the PDN. File 2 must have the same number of mass signals arranged in the same order as File 1 and its first column must be IDs. File 1 and File 2 must have identical IDs. All datasets uploaded must be tab-separated.txt or.dat files.

### Perform mass decomposition

By default, OligoNet uses 19 proteinogenic amino acids as subunits of mass decomposition (instead of 20) because of the two ambiguous by mass (Leucine/Isoleucine). Users could customize the subunits in File 3 if users do not want to use the default 19 common amino acids or wish to include non-standard amino acid/post-translational modifications. We note that the first line of the file must be the molecular weight loss of the condensation reaction (by default the loss of a H_2_O molecule). The two columns of the file should be the names or symbols of the subunits and their corresponding neutral exact mass. It is compulsory to specify the tolerance window (in Dalton) of annotation, with 0 if you have theoretical exact masses. The annotated UAAC and MAAP signals, along with absolute annotation errors (in ppm), are displayed in the tab-panels “Mass decomposition-UAAC-Annotation” and “MAAP-Annotation”. They can be also downloaded as a data matrix in a “.txt” file, where each row corresponds to the decomposition results of each mass feature. Compared to the third-party server DECOMP, output of OligoNet is more easily combined with other information about the mass features (statistical analysis, retention time, etc), and is more adapted to metabolomics studies.

The default implementation for mass decomposition (the R recursive function) is recommended for datasets that contain fewer than 500 mass signals. Otherwise, if users prefer to request the third-party server DECOMP, the checkbox “Use DECOMP server” needs to be ticked. Users could also perform the mass decomposition directly on their website (https://bibiserv2.cebitec.uni-bielefeld.de/decomp). During the data submission, the checkboxes “chemically plausible decompositions” and “actual mass for each decomposition” must be disabled and “deviation from query mass for each decomposition” must be enabled. OligoNet could then display UAAC and MAAP annotations and perform further analysis based on the DECOMP job id.

### Network construction and visualization

The PDN is a directed acyclic graph (DAG) *G = (V, E)* in which the set of vertices, *V*, represents annotated UAAC signals, and the set of edges, *E*, represents “degraded amino acids”, or the amino acid differences (ΔAA) between UAAC annotations. During network reconstruction, a polynomial-time algorithm performed an exhaustive search in a descending order across the list of UAAC annotations by calculating ΔAA between those annotations. Two vertices are connected (e.g. T1L2E1 → T1L1) only if they have an entirely positive ΔAA (e.g. ΔAA = L1E1). Users could simplify the PDN in four ways: i) by removing unconnected nodes; ii) by deleting triangles in the DAG, e.g., if both T1L2E1 → T1L1 and T1L1 → L1 are found in the DAG, the edge T1L2E1 → L1 will be removed as it might be equivalent to the chain reaction T1L2E1 → T1L1 → L1, suggested only for very complex networks; iii) by removing free amino acids from the graph since these high degree vertices could remarkably increase the network complexity; iv) by keeping only the edges that connect two highly correlated vertices according to Spearman correlation coefficient. In fact, the high correlations indicate a strong biochemical dependence between two network nodes^31^. An interactive network visualization is provided once the network is built. Users could export a Cytoscape-compatible^32^ network file for a visualization outside OligoNet. Network visualization and topological analyses are available in the tab-panel “Network results” (details in the Results session).

### Oligopeptides in the metabolic network

All mass signals in File 1 are also annotated in the KEGG compound database (http://www.genome.jp/kegg/compound/). The compound IDs of annotated peptides are available in the “.txt” file along with peptide annotations. Furthermore, OligoNet displays in the tab-panel “Peptides in KEGG” metabolic pathways in which these peptides are involved for an organism chosen. The peptides are labeled in red while other annotated metabolites in the same pathway are labeled in blue (Figure S1). This function unravels the relation between oligopeptides and other metabolites in the data.

## Results

### Annotation of yeast LMW peptidome

To illustrate the results of a typical OligoNet job, we use a previously published dataset derived from the extracellular metabolic profiles (exo-metabolome) of 15 yeast *Saccharomyces cereviesae* strains^12^. Mass spectra of 45 grape wines fermented by yeast (biological triplicates for each strain) were acquired in positive full scan mode on a Bruker solariX 12 Tesla FT-ICR-MS (Bruker Daltonics, Bremen, Germany). With a time domain of 4 megawords in the 100–1 000 m/z range, the resolving power of the mass spectra reached 400 000 at m/z = 400 and the internal calibration error was under 0.1 ppm. After spectra alignment and elemental formula annotation using the mass difference network (MDiN) approach^27, 33^, the dataset uploaded to OligoNet contains 18253 neutral theoretical masses detected in 45 samples (m/z values and intensities).

In this study, we are not interested in the possible sequences of each peptide, but rather in how each mass can be decomposed into “monomers”. Therefore, to simplify the DECOMP algorithm, glutamine (Q) and asparagine (N) were removed from the 19 default subunits for not being monomers: the glutamine is a dimer consisting of one glycine (G) and one alanine (A), while the asparagine (N) a dimer of glycines (G). With the new 17 amino acid settings, the DECOMP algorithm has annotated in total 602 UAAC and 1 004 MAAP signals in the dataset. Such a high amount of putative annotations unraveled an unprecedented diversity of oligopeptides in the yeast exo-metabolome. Among all 1 606 annotated mass signals, only 98 (free amino acids included) were also annotated in the KEGG database. In this study, the mass decomposition had a clear advantage in revealing the LMW peptidome richness over database annotation: putative annotations obtained by mass decomposition are extremely diverse in terms of peptide length and amino acid composition. Focusing on UAAC signals, we observed 4 free amino acids, 75 dipepetides, 225 tripeptides, 195 tetrapeptides, 74 pentapeptides, 28 hexapeptides and one heptapeptide. All 17 residues occurred in the annotations, and proline, glycine, leucine and cysteine were the most frequent residues (Figure S2A).

### Characteristics of the peptide degradation network

**Figure 1:**
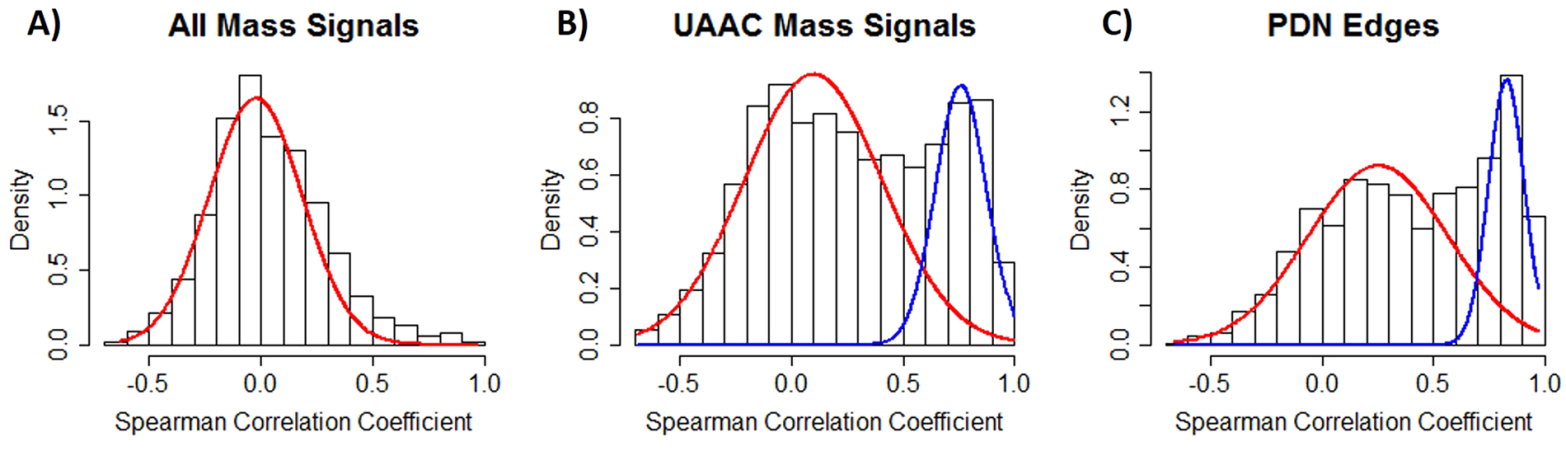
Distribution of pairwise spearman correlation coefficients (**A**) between 2 134 randomly selected mass pairs in FT-ICR-MS data, a Gaussian curve *N* (µ = 0, σ = 0.2) (the red curve) can be fitted to the distribution, (**B**) between 2 134 randomly selected UAAC mass pairs, the data can be fitted with a mixture of two Gaussians curves: *N*_1_(µ = 0.1, σ = 0.3) (the red curve) and *N*_2_ (µ = 0.8, σ = 0.1) (the blue curve) (**C**) only between connected nodes in the PDN, the data can be fitted with a mixture of two Gaussians curves: *N*_1_ (µ = 0.3, σ = 0.3) (the red curve) and *N*_2_ (µ = 0.8, σ = 0.1) (the blue curve).

The PDN is a hypothetical metabolic network that collects all potential hydrolysis reactions between annotated peptides. The PDN building is merely based on the metabolic profile, a static snapshot of physiology of the cell. As for other metabolomic data-driven networks, we need to evaluate the potential of the PDN to allow unbiased and comprehensive studies of peptide degradation systems. Indeed, the behavior of well-known complex biological systems usually emerges from the pairwise dependence between its components^34^. In the PDN, such dependence can be proved through statistical correlations between its nodes. The PDN that we evaluated was built from UAAC signals of the yeast metabolomics dataset. Free amino acids were removed since they have a strong influence on the network topology by connecting to most UAAC signals, and might lead to biased interpretations. Spearman correlation coefficient *ρ* was calculated for each connection of the PDN, and the density plot of 2 134 correlation coefficients was compared to random mass pairs selected from the entire FT-ICR-MS dataset and from all UAAC annotations (Fig. 1). We observed that the correlations in the MS dataset was clearly a zero-centered Gaussian (Fig. 1A). When we sampled only from UAAC signals, a second Gaussian distribution appeared at *ρ* = 0.8 (Fig. 1B). This distribution was more pronounced for the correlations of PDN edges (Fig. 1C). These comparisons proved the biochemical dependence between annotated peptides, and even stronger dependence between PDN connected components. To further explore the biological details of the PDN and origins that contribute to its formation, we performed a series of topological analysis on the yeast PDN. To make sure that the data-generated PDN underlies biochemical pathways, we only kept edges that connect two highly correlated vertices (*ρ* ≥ 0.8).

**Figure 2:**
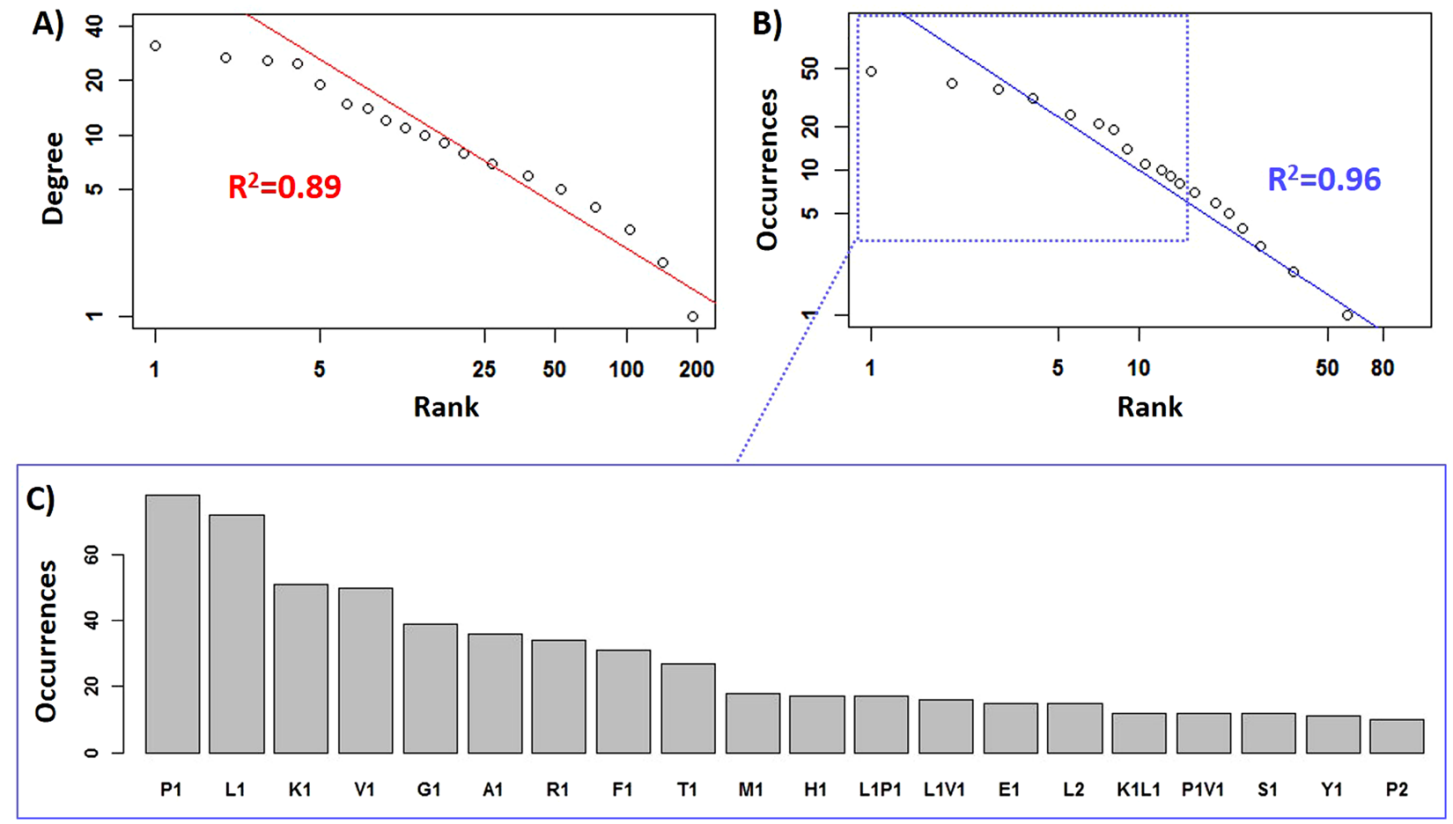
Topological analysis of FT-ICR-MS-based yeast PDN: (**A**) Zipf plot of vertex degree (sum of in-degree and out-degree) as a function of its rank, (**B**) Zipf plot of occurrences of a specific reaction (peptide degradation) as a function of its rank, (**C**) the 20 most frequent reactions in the yeast PDN. The fitted lines in (**A**,**B**) are both based on power-law distribution.

Topological studies showed a strong similarity between simplified yeast PDN and actual metabolic networks. Firstly, the PDN shows a “scale-free” topology, i.e. vertex degree depends on its rank in the form of a power-law (Fig. 2A). This means that there are few high-degree nodes, known as metabolic “hubs”, but a large majority of nodes have low degree. Such a distribution would not be expected in a random network, but has been found typical for metabolic networks^34, 35^. The highly-connected nodes could play key roles in a biological system^34^. In the PDN, they can be interpreted as either the final products of peptide degradation or common amino acid combinations of several longer peptides. For instance, the vertex “P1F1” has an in-degree of 10 and it is the “common pattern” of 10 UAAC signals such as P1F1R1, P1V1F1 (Figure S3A). Interestingly, it is reported that peptides that contain a proline residue contiguous to the phenylalanine could bring a strong bitter taste to food products^36^. In fact, it has been reported that the oligopeptides with common fragments in their sequence or rich in certain amino acids have similar biological functions and the mixture of these peptides will have a synergistic effect^37, 38^. Accordingly, we found several literature-reported common patterns in the yeast PDN, including the bitter taste “P1R1”^38^, antioxidant “L1P1”^39^, and antihypertensive “A1P1”^40^ and “A1F1”^41^. The PDN also contains a few high out-degree nodes (Figure S3B), that can be considered as starting points of degradation reactions. Highly-connected network vertices and their surrounding nodes can be visualized in the tab-panel “Network Results-Subgraphs”.

Further confirmation of the metabolic network nature of PDN is provided by an analysis of the frequency of various amino acid(s) loss (edges of PDN). As shown in Fig. 2B, the number of times a specific amino acid(s) loss is observed depends on its rank in the form of a power-law (Fig. 2B). Such a distribution was also mentioned in various metabolic networks^35, 42^. Our hypothesis is that a few principal reactions (Fig. 2C), along with many rare amino acid(s) loss, could give birth to the diversity of LMW peptidome. The fact that some reactions occurred much more frequently might be related to amino acid-specific peptidases released by yeast during alcoholic fermentation^43^.

Another interesting property of the yeast PDN is that it contains long paths (>2 edges), e.g., P1V2L2 → P1V1L2 → P1V1L1 → P1V1. These long paths, displayed in the tab-panel “Network Results-Subgraphs”, can be interpreted as “snapshots” of peptide stepwise degradation. It’s worth studying these chain reactions since the stepwise breakdown of longer protein fragments could produce important bioactive oligopeptides^44^. Interestingly, all 48 long paths found in the PDN start with one of the three vertices: “P1V2L2”, “P1V1L2K1” or “G1A1K1F1R1” (Figure S3C). It seems that these specific peptides “initiates” the chain reactions. Further investigation is required to understand the biological nature of these peptides.

### Results of OligoNet on a lower resolution metabolomics dataset

OligoNet was tested on a lower resolution metabolomics dataset. Same 45 wine samples were studied on a TOF-MS system (Synapt HDMS aoQTOF, Waters, Milford, MA) coupled to the ACQUITIY UPLC (Waters, Milford, MA). The reversed-phase (RP) gradient and positive ionization mode MS settings for LC-MS metabolic profiling was described in Liu *et al*.^12^. The resolving power and mass accuracy of the TOF-MS was 10 000–50 000 and 1 ppm to 5 ppm, respectively. Metabolic features were extracted based on their mass and retention time (RT) pairs after spectra alignment. Isomers with the same mass and different RTs were considered as separate features. We used experimental neutral masses for mass decomposition with default subunit settings. With a tolerance window of 0.001 Da, 480 out of 7 608 metabolic features (RT-mass pair) were annotated as UAAC signals and 770 as MAAP signals. As in FT-ICR-MS data, leucine, proline, glycine and cysteine were the most frequent residues among UAAC annotations (Figure S2B). Such agreement between two analytical platforms indicates clearly a non-randomness of our peptide annotation procedure.

**Figure 3:**
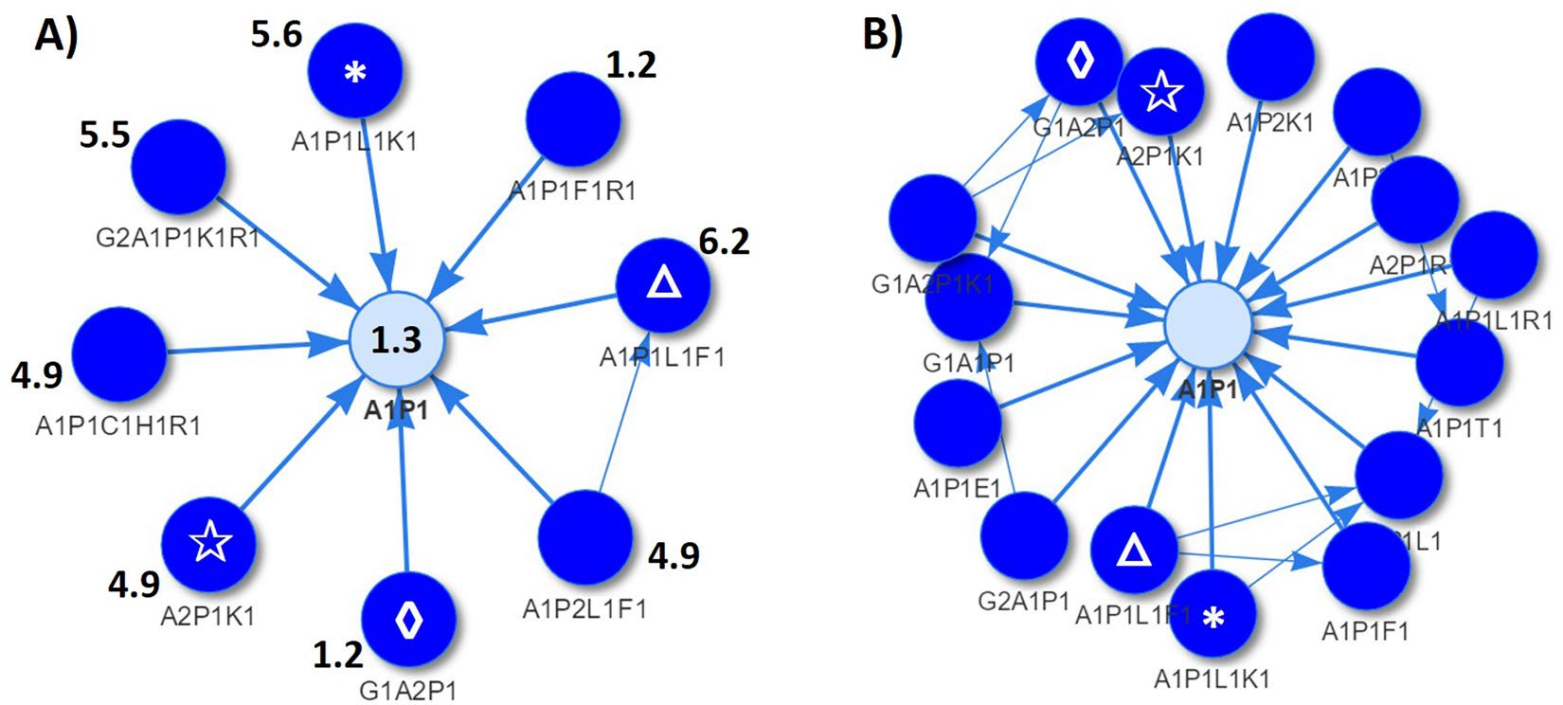
(**A**) The highly-degree vertex “A1P1” and its surrounding nodes in the LC-MS-based yeast PDN. The number next to each node is the retention time of the corresponding molecular feature. (**B**) The high in-degree vertex “A1P1” and its surrounding nodes in the FT-ICR-MS-based yeast PDN. The manually-added symbols Δ, *, ✰ and ◊ in (**A**,**B**) indicate the same surrounding nodes that are found in both networks.

Likewise, we built a free amino-acid removed PDN based on annotated UAAC metabolic features. Interestingly, statistical and topological analysis of the LC-MS-based PDN showed comparable results to FT-ICR-MS-based PDN. Firstly, the distribution of Spearman correlation coefficients between paired nodes was a mixture of two Gaussian distributions centered at 0 and 0.8, respectively. By applying the correlation filter *ρ* ≥ 0.8, we observed the similar vertex degree distribution thereby the “scale-free” topology (Figure S4A compared to Fig. 2A). The edge frequency also followed a power-law distribution and the four most frequent reactions were L1, V1, P1 and K1 as in FT-ICR-MS data (Figure S4BC compared to Figure S2BC, without counting the edges between isomeric features). We found several high-degree vertices that occurred in both LC-MS and FT-ICR-MS-based PDN, such as the antihypertensive common pattern “A1P1” (Fig. 3). Due to the lower resolution and lower sensitivity, “A1P1” was surrounded by fewer nodes in LC-MS data. Interestingly, among 8 surrounding nodes in Fig. 3A, 4 of them were also detected in Fig. 3B. In fact, we observed a good agreement of surrounding nodes between FT-ICR-MS and LC-MS-based PDN for most high-degree common patterns. For LC-MS data, RT of each metabolic feature allowed generating more hypotheses about detected peptides since the retention behavior of peptides in the RP-UPLC is affected by their amino acid composition, peptide chain length and sequence^45^. Some connected vertices in PDN clearly reflected these influencing factors. For instance, the node “A1P1” has considerably lower retention time than 6 out of 8 surrounding longer peptides that contain “P1L1” and other residues (Fig. 3A).

In summary, OligoNet reflects the chemical diversity of LMW peptidome from yeast metabolomics data with lower mass resolution. Despite much lower mass accuracy (because of the TOF-MS and the fact that the masses were not annotated), hypotheses generated by PDN seem to be comparable and consistent between FT-ICR-MS and LC-MS data for the same set of samples.

### Results of OligoNet compared to publicly-available databases

To validate the potential biological meaning of OligoNet annotations, metabolomics datasets were additionally annotated by the bioactive peptide database PeptideDB^46^. We present here the results of two additional example datasets “Positive_Doing.txt” and “Wine_data.txt” available on the OligoNet server. 18 and 12 masses were assigned by PeptideDB from Doping and Wine, respectively. All 30 masse were annotated by OligoNet, and the amino acid compositions suggested by OligoNet were in concordance with the database results (Table S2). This validation confirms the potential of OligoNet to discover bioactive peptides.

To give a more rigorous validation of how precisely OligoNet could annotate real peptides, we extracted randomly 467 masses from the publicly available HeLa label-free proteomics dataset^47^ (identifier: PXD000612 on proteomexchange). All masses in this dataset were annotated by the human Uniprot FASTA database along with their sequences. Before importing this mass list to OligoNet, a random error of an amplitude of 0.01 Da was added to each exact mass to mimic the instrumental mass deviations. Despite the added error, all 467 masses were annotated by OligoNet, and the amino acid compositions of all 467 masses suggested by OligoNet were in agreement with the peptide sequence deduced by the standard proteomics approach (Table S3). These findings indicate that OligoNet is sensitive to detect peptide features and could provide reliable annotations for experimental mass signals.

## Discussion

**Figure 4:**
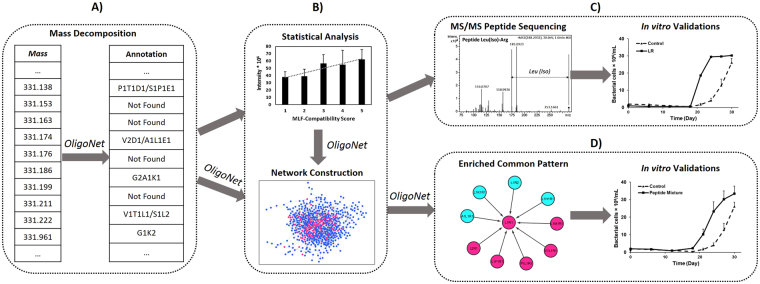
Characterization of yeast-derived peptides involved in MLF-stimulation using the web server OligoNet (**A**) Mass features that could be decomposed into a combination(s) of amino acids were considered as potential peptides. (**B**) Each potential peptide was evaluated statistically for biomarker discovery. Discriminant features were extracted if both conditions were filled: (i) the averaged intensity of each strain and MLF-compatibility score was correlated (spearman correlation coefficient ρ ≥ 0.4); (ii) the Kruskal-Wallis test revealed a significant difference between 5 phenotype levels by p-value < 0.1 when considering all biological replicates (without averaging intensities). (**C**) The mass signal m/z = 288.203, annotated as L1R1, was a potential biomarker according to statistical analysis. Its sequence was determined as “Leu(Iso)-Arg” based on the LC-MS/MS spectrum. A time-course *in vitro* validation was started by adding 0.9 μmol Leu-Arg standard into 15-ml bacterial culture. Compared to the control culture, an accelerated bacterial growth from T = 18 days was observed. Experimental details about the bacterial culture can be found in Liu *et al*.^12^. (**D**) In the PDN, a local enrichment of potential MLF-stimulatory peptides was observed in the “L1R1” common pattern: 6 out of 10 nodes showed statistical discrimination (discriminant nodes are colored in pink). To confirm the function of the pattern observed, a mixture of four peptides was added into the 15-ml bacterial culture: Leu-Arg, Phe-Ser-Leu-Leu-Arg-Asn, Tyr-Gly-Gly-Phe-Leu-Arg and Arg-Leu-Arg-Phe-Asp (0.23 μmol each). An enhanced bacterial growth after the lag phase was observed.

To illustrate how the web server could add values into an untargeted metabolomics study, a workflow for biomarker discovery using the above-mentioned FT-ICR-MS-based yeast metabolic profiling data is provided (Fig. 4). Aiming at discovering new yeast-derived peptides involved in the stimulation of bacteria-driven malolactic fermentation (MLF), this study is a continuation and extension of the work described in Liu *et al*.^12^. In this earlier work, metabolic profiling was performed for 15 yeast strains (S1–S15) in triplicates. These strains held reproducible MLF-compatibility scores (from 5 to 1, 5 being the most compatible, 1 being the least compatible). The FT-ICR-MS dataset was statistically evaluated to extract mass features whose level was positively correlated with MLF-compatibility scores. Liu *et al*. has suggested oligopeptides as an important family of MLF-stimulatory compounds via elemental formula and database annotation of these discriminant features.

To further characterize the biochemical nature of potential LMW peptides, the web server was used in the current workflow to allow: i) a fast identification of potential peptides among discriminant features (Fig. 4C); ii) the association of specific subnetwork of PDN with MLF stimulation (Fig. 4D). Based on i), we performed LC-MS^2^ experiments on discriminant masses that could be decomposed into combination(s) of amino acids, and putative structures of some MLF-stimulatory peptides were determined via *de novo* sequencing (Figure S5 and Table S1). We confirmed that the results of *de novo* sequencing agreed with their annotated amino acid combinations (Table S1). In addition to statistical (Fig. 4B) and structural (Fig. 4C) validation of peptide biomarkers, we performed biological validation by supplementing a bacteria culture with synthetic peptide standards according to the sequencing results. The use of web server in the workflow enabled us to discover and validate novel MLF-stimulatory compounds, such as Leu-Arg (Fig. 4C).

Moreover, combining the results of statistical analysis, the visualization of PDN on the web server led to specific network zones that could be involved in the MLF-stimulation (Fig. 4D). Interestingly, nodes that represent discriminant mass features appeared to be abundant in some of above-mentioned common pattern regions (high in-degree “hubs” and their surrounding nodes) (Figure S6). These network regions, statistically related to MLF-stimulation, might represent a mixture of potential biomarkers with common fragments in their sequences. As for the “L1R1” common pattern region, we further validated whether a mixture of 4 random peptides that contained “Leu-Arg” in their sequence could have a physiological impact on bacteria as individual peptides (Fig. 4D). The mixture solution used for supplementation was made to provide the same amount of peptides available for bacteria as in Fig. 4C, and the growth stimulation effect was still observed. Our results showed that the peptide function (here MLF-stimulation) could be related to certain fragments in their sequences, and the system biology approach provided by the web server can definitely ease the investigation of such relationship. On the other hand, the common pattern region in Fig. 4D consists of nodes, such as A1L1R1 and L1K1R1, that are not related to MLF-stimulation. Compared to MLF-related peptides, the difference in amino acid composition (other than Leu and Arg) might explain the difference in peptide function. The network analysis is useful here to display those property differences that require further investigation.

## Conclusion

OligoNet is a web-service that facilitates the extraction of potential oligopeptides from untargeted metabolomics datasets. After a MS full scan experiment, OligoNet provides a rapid, database-free solution to find the amino acid composition of all potential peptides under 1 500 Da. However, the main purpose of the software is the biological analysis of oligopeptides with 3–8 residues (300–900 Da) for which little information is available in metabolomics or proteomics databases. Our software was designed to provide more information about them and to fill in this blank space of databases.

The software is adapted for both ultrahigh resolution and high resolution direct infusion or LC/GC-MS, and for both annotated and original MS data. Considering that no specific peptide enrichment or separation techniques were introduced, the outputs of OligoNet on two yeast metabolic profiling datasets unraveled an unprecedented diversity of oligopeptidic features including both known and unknown peptides. These results showed the potential of untargeted metabolomics for the characterization of LMW peptidome, and moreover, the discovery of oligopeptidic biomarkers through the combination of statistical analysis and the use of the web server.

In addition, OligoNet generates an *ab initio* network that describes the connections between annotated peptides. The generation of PDN differs fundamentally from other data-driven network reconstruction approaches^27, 35^ in three ways: i) solely on putative annotations; ii) only focusing on one type of reaction (peptide degradation) between one family of compounds (peptides); iii) no specific transformation list is required. Interestingly, the topology and the statistical properties of yeast PDN were very close to a reconstructed metabolic network, and were reproducible on both metabolomic platforms. That’s why multiple biochemical hypotheses can be generated from specific sub-networks such as the high-degree vertices and long network paths. From these detected structures, or “patterns”, some potential functions of the LMW peptidome can be inferred based on statistical analysis (e.g. p-values of vertices in the pattern) and literatures (such as the antihypertensive “A1P1”^40^). Also, we have shown in the manuscript the possibility and a workflow to validate biologically the observed patterns.

OligoNet also unravels the connections between peptides and other detected metabolites in a selected metabolic pathway. In future studies, it is possible to relate a confirmed PDN pattern with other important metabolic pathways. This new approach would allow a better understanding of the LMW peptidome in system biology, and could be applied to other classes of compounds with repeating units such as sugar, lipid and polyphenol. These functions will be implemented in future versions of OligoNet.

## Electronic supplementary material

Supplementary Figures and Table S1

Supplementary Table S2

Supplementary Table S3
