# Supplementary material for: Digging into the low molecular weight peptidome with the OligoNet web server
Source: Sci Rep. 2017 Sep 15;7:11692. doi: 10.1038/s41598-017-11786-w (PMC5601033; doi:10.1038/s41598-017-11786-w)
Supplement: Supplementary file 1 — Supplementary Figures and Table S1 [file 41598_2017_11786_MOESM1_ESM.doc]

**Supplementary figures and tables to** «**Digging into the low molecular weight peptidome with the OligoNet web server**»

Youzhong Liu^1^, Sara Forcisi^2^, Marianna Lucio^2^, Mourad Harir^2,3^, Florian Bahut^1^, Magali Deleris-Bou^4^, Sibylle Krieger-Weber^4^, Régis D Gougeon^1^, Hervé Alexandre^1^ and Philippe Schmitt-Kopplin^2,3*^

^1^UMR PAM Université de Bourgogne/Agrosup Dijon, Institut Universitaire de la Vigne et du Vin, Jules Guyot, Rue Claude Ladrey, Dijon, France

^2^Research Unit Analytical BioGeoChemistry, Department of Environmental Sciences, Helmholtz Zentrum München, Ingolstädter Landstr.1, Neuherberg, Germany

^3^Technische Universität München, Chair of Analytical Food Chemistry, Freising-Weihenstephan, Germany

^4^Lallemand SAS, 19 rue des Briquetiers, Blagnac, France

*schmitt-kopplin@helmholtz-muenchen.de


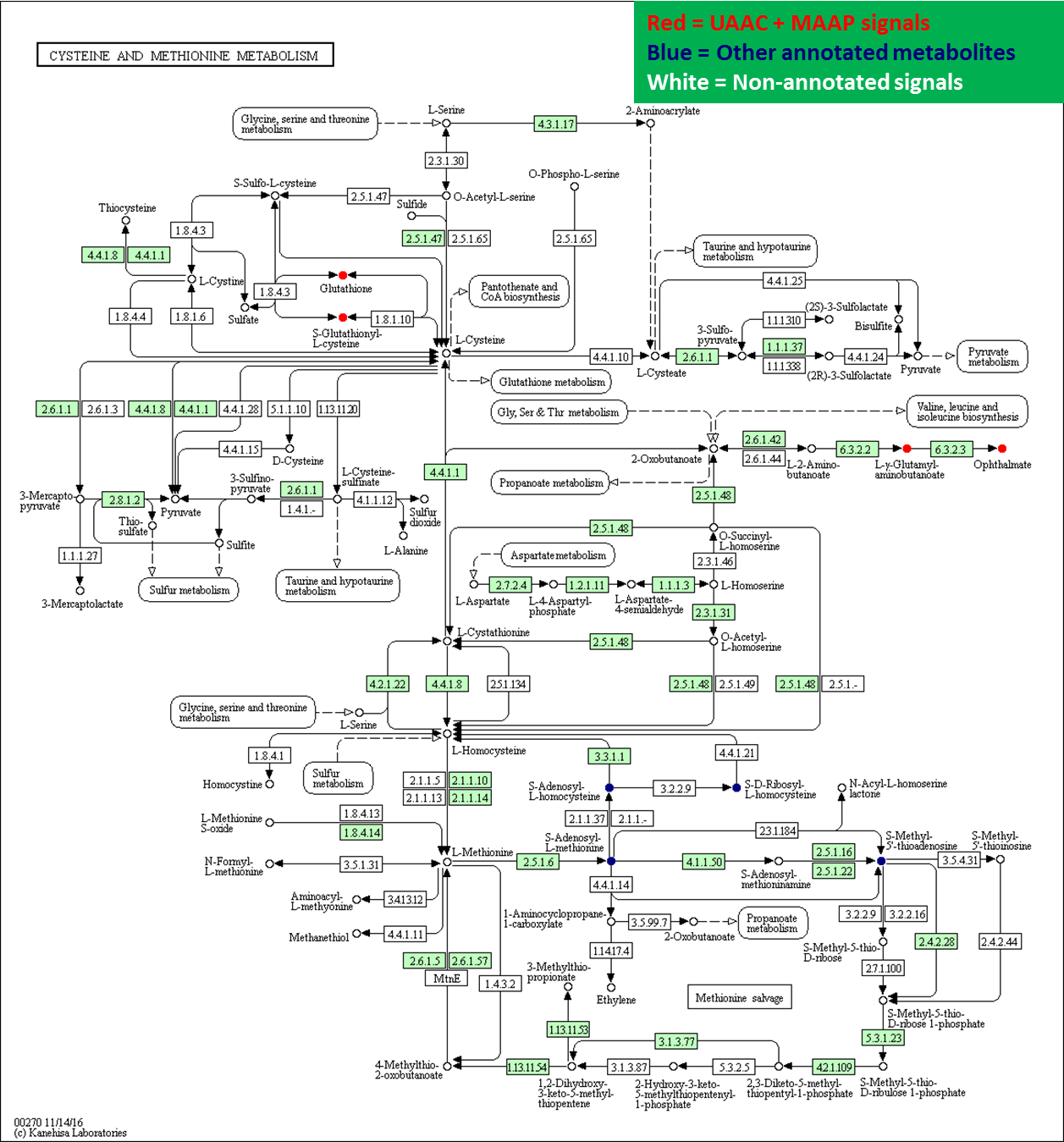


**Figure S1** Visualization of *Saccharomyces cereviesae* pathway "CYSTEINE AND METHIONINE METABOLISM" in the KEGG Database (http://www.kegg.jp/kegg/) (1). The nodes represent metabolites, the arrows represent biochemical reactions and the rectangles represent enzymes. Red nodes and blue nodes are respectively putative peptides (UAAC + MAAP masses) and other annotated metabolites.


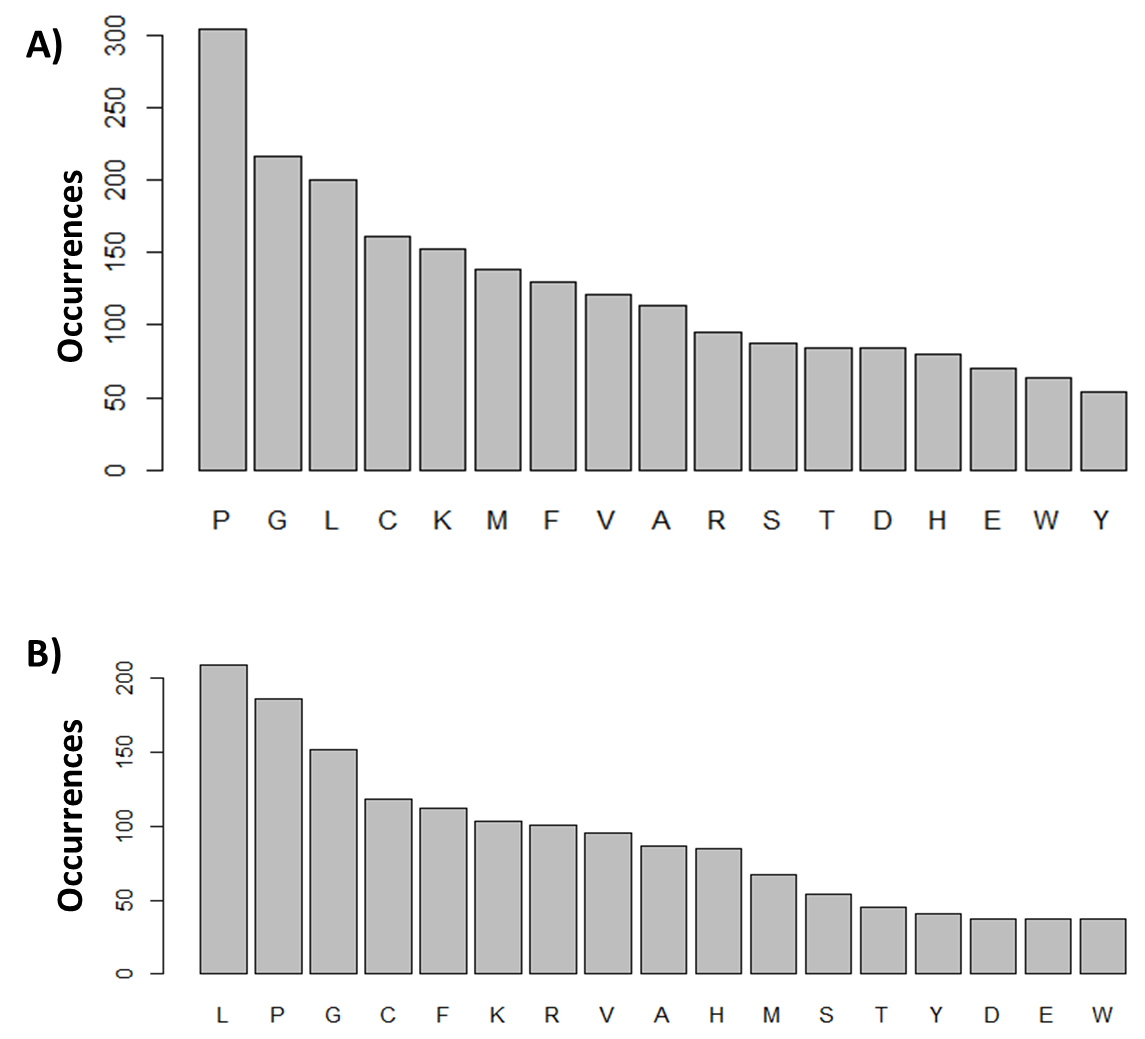


**Figure S2** Occurrences of different amino acids across all UAAC annotations of A) FT-ICR-MS data, B) LC-MS data. Following example shows how the calculation was performed for one UAAC annotation: in the annotation "P2C2K2", proline, cysteine and lysine all appear twice.

.


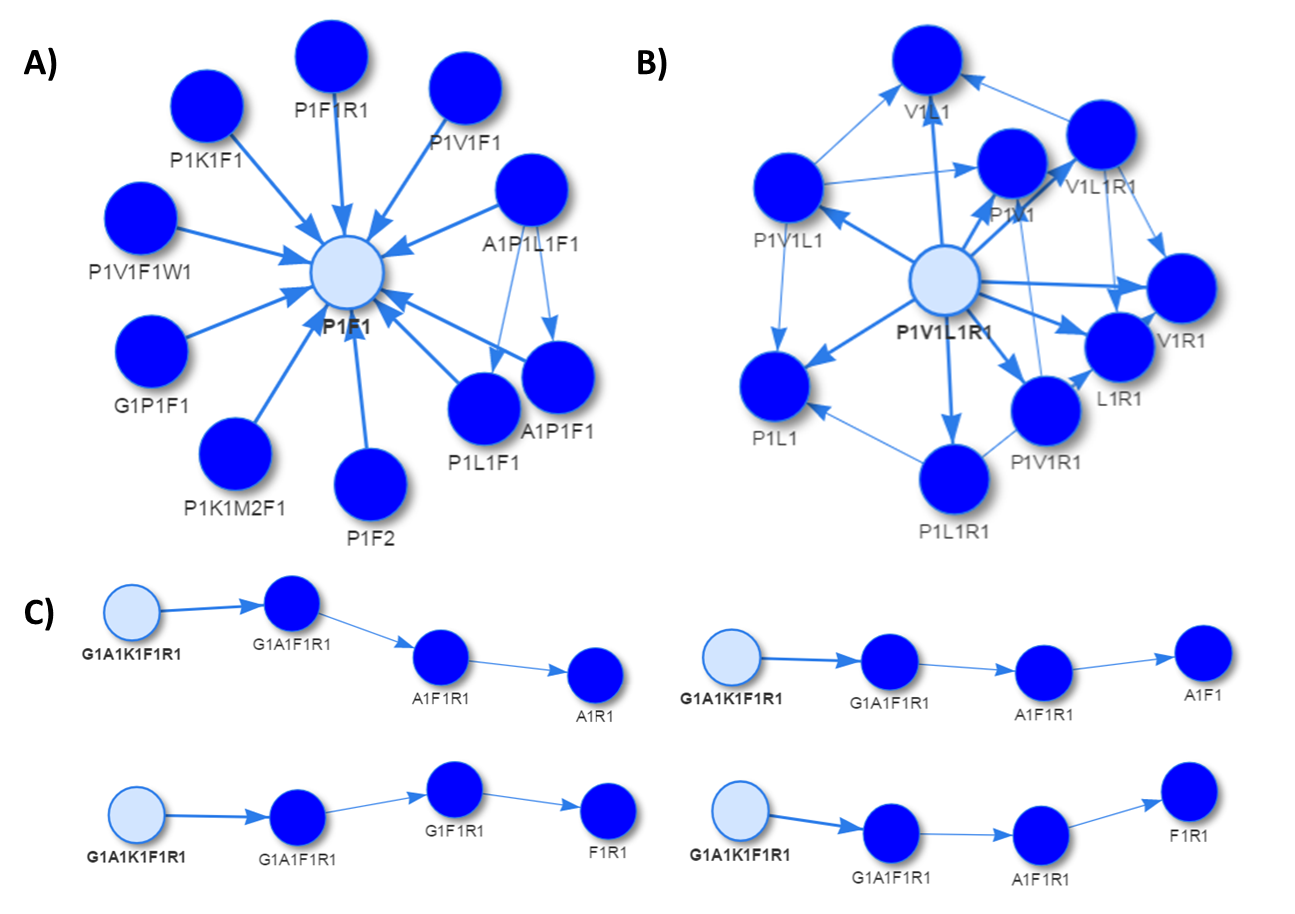


**Figure S3** Subgraphs of a yeast PDN: A) the highly-connected vertex "P1F1"; B) the high out-degree vertex "P1V1L1R1"; C) long paths (length = 4), all of them start with "G1A1K1F1R1".


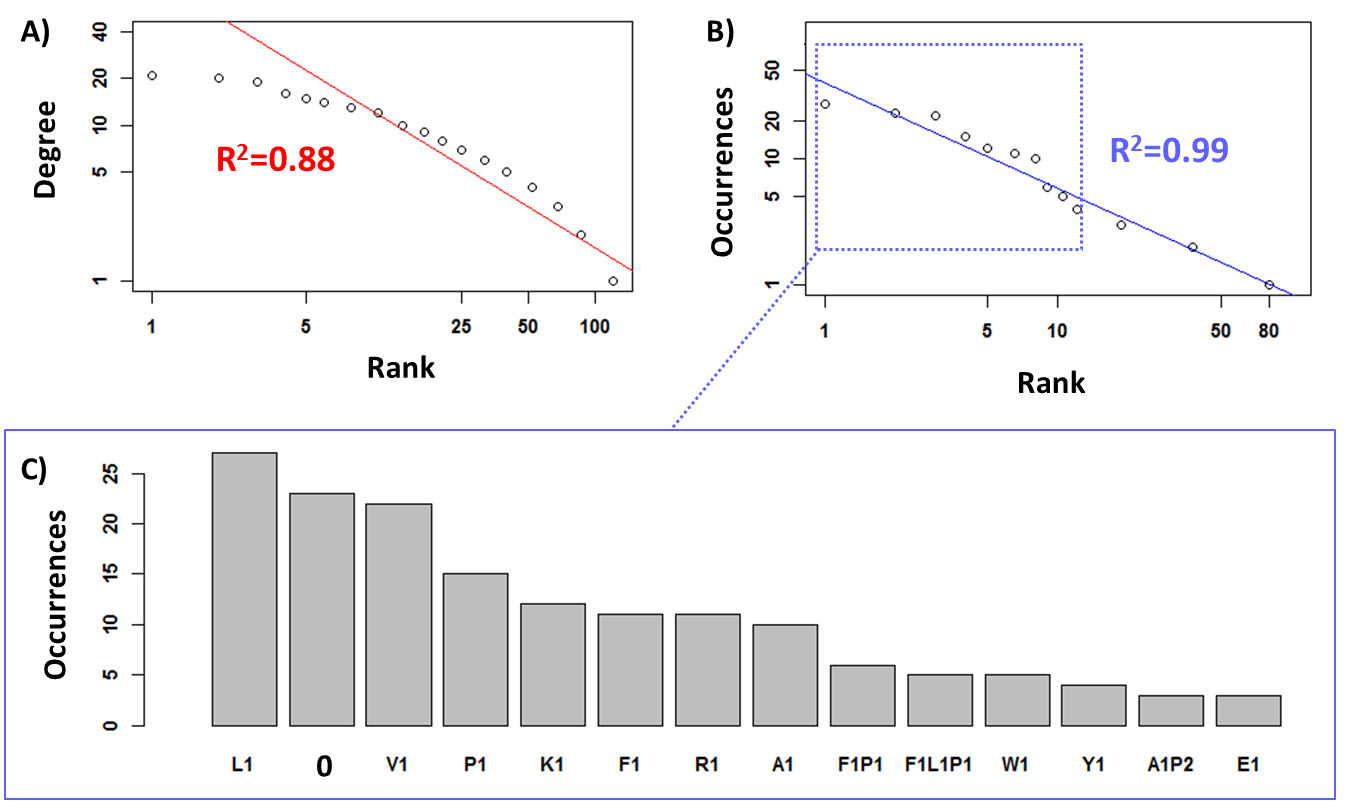


**Figure S4** Topological analysis of LC-MS-based yeast PDN: A) Zipf plot of vertex degree (sum of in-degree and out-degree) as a function of its rank; B) Zipf plot of occurrences of a specific reaction (peptide degradation) as a function of its rank. The fitted lines in A) and B) are both based on power-law distribution. C) The 14 most frequent reactions in the yeast PDN. The reaction "0" here represents the connections between two isomeric features.


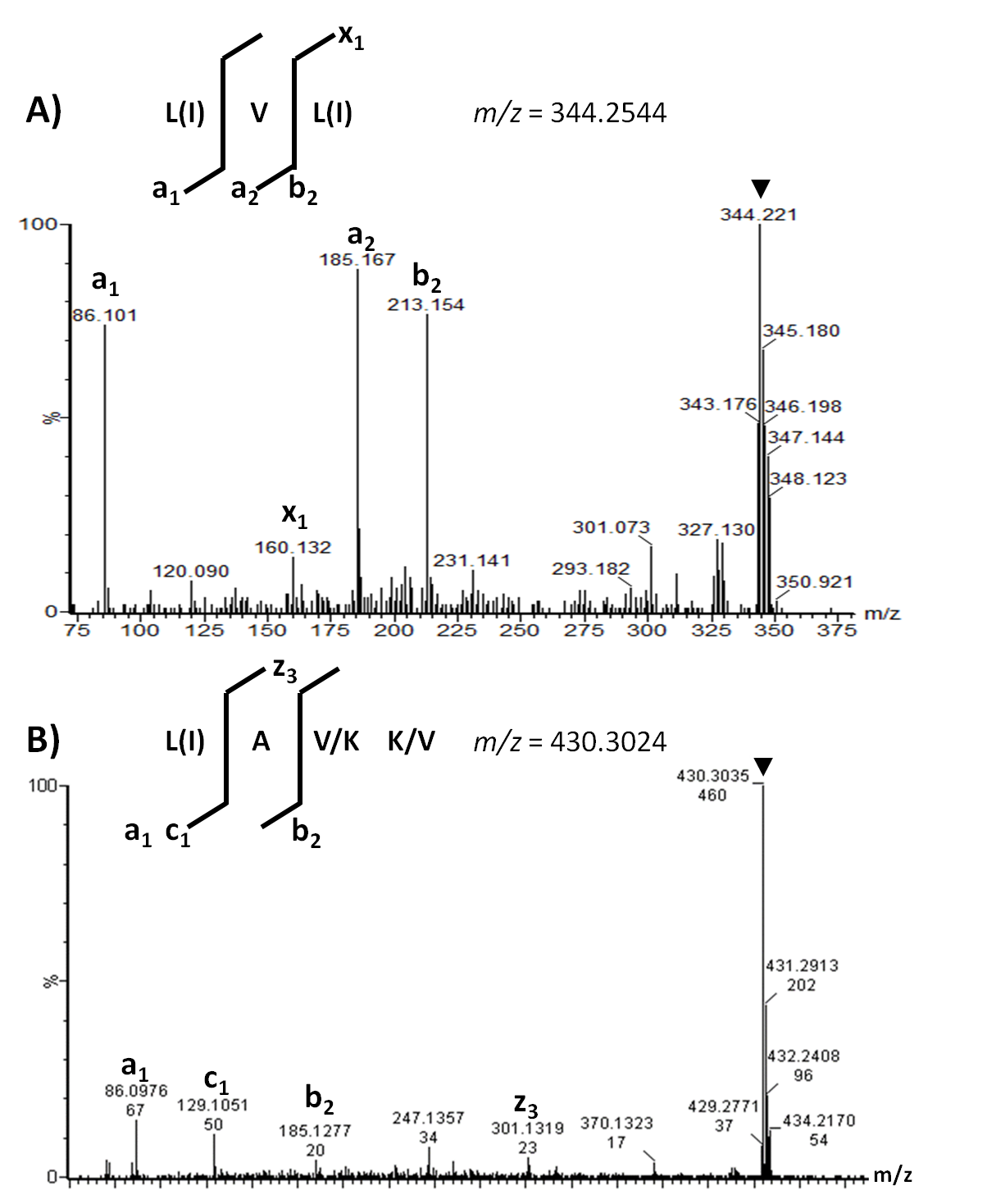


**Figure S5** Structures of some OligoNet-annotated MLF-stimulatory signals (non-annotated in KEGG compound database) were confirmed by LC-MS^2^ experiments with TOF-MS (Synapt HDMS aoQTOF, Waters, Milford, MA) coupled to the ACQUITIY UPLC system (Waters, Milford, MA). 45 samples analyzed by FT-ICR-MS were pooled and the mixture was analyzed with reversed phase chromatography. Candidate masses were isolated and fragmented with a 20-eV collision energy. Two examples of MS/MS spectra are provided. The *de novo* sequencing was performed with METLIN server (2) (<https://metlin.scripps.edu/>) in combination with manual interpretations. A) The mass signal *m/z* = 344.2544 is annotated as a combination of two (iso)leucines and one valine by DECOMP, and its sequence is L(I)VL(I) according to the fragments. B) The MAAP signal *m/z* = 430.3024 has two possible amino acid combinations according to DECOMP: G1K1L(I)2 or A1K1L(I)1V1, and the LC-MS^2^ confirmed the second combination along with its sequence: L(I)AVK or L(I)AKV.


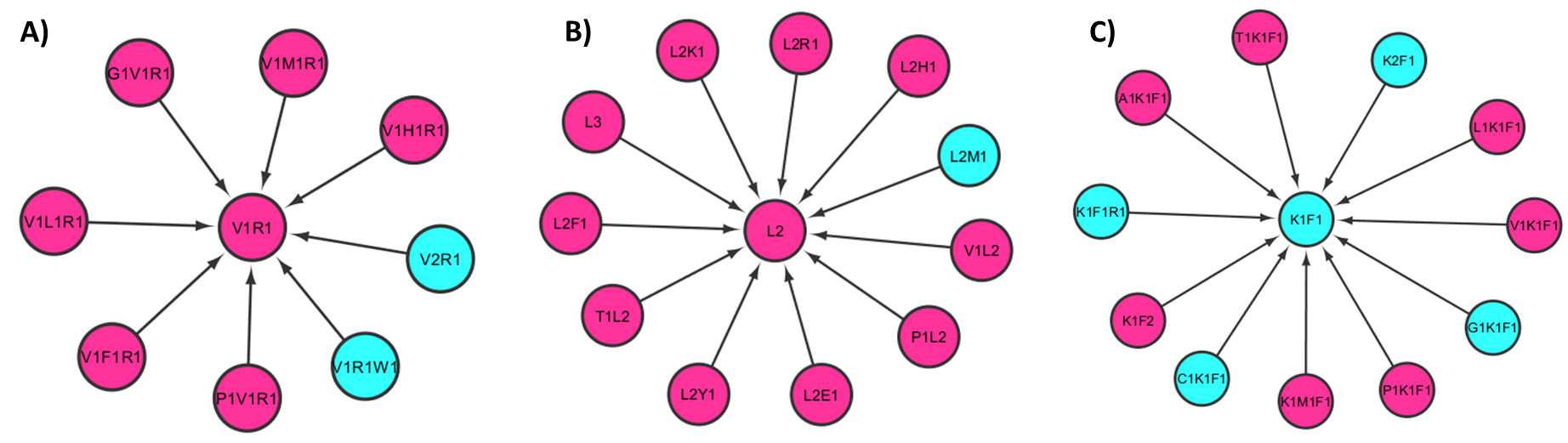


**Figure S6** Looking over the PDN built from FT-ICR-MS data, we noticed that statistically-determined peptide biomarkers (colored in pink) were particularly abundant in some of common pattern regions. For instance, we found A) 7 out of 9 biomarkers in the V1R1-common pattern, B) 11 out of 12 biomarkers in the L2-common pattern and C) 7 out of 12 biomarkers in the K1F1-common pattern. These percentages showed a considerable local enrichment of biomarkers in these PDN regions, knowing that only 112 out of 587 nodes PDN represent potential biomarker.

**Table S1** LC-MS/MS sequencing results of potential MLF-stimulatory biomarkers along with results of the statistical analysis (column “Cor”: the correlation between MLF-compatibility and intensity of the mass signal across the strains; column “P-value”: statistical significance of the Kruskal-Wallis test).

| ***m/z*** | **Cor** | **P-value** | **OligoNet Annotation** | **Product ions (*m/z*)** | **Sequence** |
| --- | --- | --- | --- | --- | --- |
| 344.2544 | 0.53 | 0.03 | V1L2 | 86.1 (a_1_), 160.13 (x_1_), 185.17 (a_2_), 213.15 (b_2_) | Leu(Iso)-Val-Leu(Iso) |
| 430.3024 | 0.61 | 0.09 | G1L2K1/A1V1L1K1 | 86.1 (a_1_), 129.11 (c_1_), 185.13 (b_2_),301.1 (z_3_) | Leu(Iso)-Ala-Val-Lys/ Leu(Iso)-Ala-Lys-Val |
| 288.2032 | 0.49 | 0.03 | L1R1 | 158.09 ([175.12-NH_2_]^+^), 175.12 (y_1_) | Leu(Iso)-Arg |
| 245.1860 | 0.58 | 0.03 | L2 | 132.1 (y1), 199.18 ([M-COOH]^+^) | Leu(Iso)-Leu(Iso) |
| 215.1391 | 0.53 | 0.02 | P1V1 | 112.08 (c_1_), 116.07 (y_1_), 156.07 (x_1_) | Val-Pro |
| 346.1973 | 0.45 | 0.05 | V2E1/V1L1D1 | 185.16 (a_2_), 213.16 (b_2_), 233.11 (y_2_) | Leu(Iso)-Val-Asp |
| 358.2700 | 0.55 | 0.01 | L3 | 199.18 (a_2_), 245.1865 (y2) | Leu(Iso)-Leu(Iso)-Leu(Iso) |
| 382.2449 | 0.59 | 0.02 | L2H | 223.16 (a_2_), 251.15 (y2) | Leu(Iso)-His-Leu(Iso) |

**Reference**

1. Kanehisa, M. & Goto, S. KEGG: kyoto encyclopedia of genes and genomes. *Nucleic Acids Res.* **28**, 27–30 (2000).

2. Smith, C. A. *et al.* Metlin: A metabolite mass spectral database. *Drug Monit* **27,** 747–751 (2005).
